# Supplementary material for: Graded heterogeneity of metabotropic signaling underlies a continuum of cell-intrinsic temporal responses in unipolar brush cells
Source: Nat Commun. 2021 Oct 7;12:5491. doi: 10.1038/s41467-021-22893-8 (PMC8497507; doi:10.1038/s41467-021-22893-8)
Supplement: Supplementary file 2 — Reporting Summary [file 41467_2021_22893_MOESM2_ESM.pdf]

nature research

Corresponding author(s): Wade Regehr

Last updated by author(s): Mar 22, 2021

Reporting Summary

Nature Research wishes to improve the reproducibility of the work that we publish. This form provides structure for consistency and transparency in reporting. For further information on Nature Research policies, see our Editorial Policies and the Editorial Policy Checklist.

Statistics

For all statistical analyses, confirm that the following items are present in the figure legend, table legend, main text, or Methods section.

n/a	Confirmed	
☐	☒	The exact sample size (*n*) for each experimental group/condition, given as a discrete number and unit of measurement
☐	☒	A statement on whether measurements were taken from distinct samples or whether the same sample was measured repeatedly
☐	☒	The statistical test(s) used AND whether they are one- or two-sided<br>*Only common tests should be described solely by name; describe more complex techniques in the Methods section.*
☒	☐	A description of all covariates tested
☒	☐	A description of any assumptions or corrections, such as tests of normality and adjustment for multiple comparisons
☐	☒	A full description of the statistical parameters including central tendency (e.g. means) or other basic estimates (e.g. regression coefficient) AND variation (e.g. standard deviation) or associated estimates of uncertainty (e.g. confidence intervals)
☐	☒	For null hypothesis testing, the test statistic (e.g. *F*, *t*, *r*) with confidence intervals, effect sizes, degrees of freedom and *P* value noted<br>*Give P values as exact values whenever suitable.*
☒	☐	For Bayesian analysis, information on the choice of priors and Markov chain Monte Carlo settings
☒	☐	For hierarchical and complex designs, identification of the appropriate level for tests and full reporting of outcomes
☐	☒	Estimates of effect sizes (e.g. Cohen's *d*, Pearson's *r*), indicating how they were calculated

Our web collection on statistics for biologists contains articles on many of the points above.

Software and code

Policy information about availability of computer code

Data collection	Commercial softwares including Igor Pro 7 (Wavemetrics) and MultiClamp 700B (Axon Instruments) were used in the collection of the physiology dataset. An Igor Pro plug-in mafPC (Matthew Xu-Friedman, SUNY Buffalo) was used for controlling the experiments.
Data analysis	Analysis were made using custom code in both Igor Pro 7 and MATLAB 2017a. Code will be made available on the primary author's GitHub repository at https://github.com/chongguo.

For manuscripts utilizing custom algorithms or software that are central to the research but not yet described in published literature, software must be made available to editors and reviewers. We strongly encourage code deposition in a community repository (e.g. GitHub). See the Nature Research guidelines for submitting code & software for further information.

Data

Policy information about availability of data

All manuscripts must include a data availability statement. This statement should provide the following information, where applicable:

- Accession codes, unique identifiers, or web links for publicly available datasets
- A list of figures that have associated raw data
- A description of any restrictions on data availability

The datasets are available from the corresponding author on reasonable request.

Field-specific reporting

Please select the one below that is the best fit for your research. If you are not sure, read the appropriate sections before making your selection.

[x] Life sciences [ ] Behavioural & social sciences [ ] Ecological, evolutionary & environmental sciences

For a reference copy of the document with all sections, see nature.com/documents/nr-reporting-summary-flat.pdf

Life sciences study design

All studies must disclose on these points even when the disclosure is negative.

Sample size	Sample size were not predetermined. Pharmacology with synaptic blockers had all-or-none effect and so a sample size equal to or larger than 5 were used in most cases. A post hoc power analysis suggest a power of 99.9% (AMPA/NMDAR antagonists, for an estimated effect size of 0.68 based on Borges Trussell, 2016), 100% (mGluR1 antagonist) and 99.3% (DGK inhibitor II) for the three pharmacology experiments in Figure 3 respectively. The use of 84 cells to extrapolate the distribution of the population behavior via linear regression achieves a power of 94%. Multiple repeats measuring the mossy fiber response of individual cells were done to resolve the shape of the temporal response curves (n=3~5). Responses are nearly identical across repeats (Supplementary Figure 1). Only a minority of cells had repeats fewer than 3 due to stability of recording.
Data exclusions	A selection criteria based on stability of response was imposed during recording. In pharmacology experiments only cells with a stable baseline response were used in subsequent analysis. Cells with unreliable MF responses across trials were excluded from analysis.
Replication	Replicates were performed across days using different animals. Pharmacology experiment were replicated in a minimum of 3 animals and results were consistent across days. DGK Inhibitory experiment were done independently by two experimenters.
Randomization	Adult animals of either sex were used. Allocation into experimental groups are completely random. All pharmacology experiments were double-blind. Covariates are controlled by balancing the coded experimental conditions by an independent lab personnel.
Blinding	All pharmacology experiment were blinded during data collection and analysis. Decoding is done only at the last stage.

Reporting for specific materials, systems and methods

We require information from authors about some types of materials, experimental systems and methods used in many studies. Here, indicate whether each material, system or method listed is relevant to your study. If you are not sure if a list item applies to your research, read the appropriate section before selecting a response.

Materials & experimental systems

n/a	Involved in the study
[x]	[ ] Antibodies
[x]	[ ] Eukaryotic cell lines
[x]	[ ] Palaeontology and archaeology
[ ]	[x] Animals and other organisms
[x]	[ ] Human research participants
[x]	[ ] Clinical data
[x]	[ ] Dual use research of concern

Methods

n/a	Involved in the study
[x]	[ ] ChIP-seq
[x]	[ ] Flow cytometry
[x]	[ ] MRI-based neuroimaging

Animals and other organisms

Policy information about studies involving animals; ARRIVE guidelines recommended for reporting animal research

Laboratory animals	Adult P30 to P40 BL6 mice of either sex used in this study were purchased form Charles Rivers. Housing conditions and other information are specified in the manuscript.
Wild animals	No wild animals were used for the study
Field-collected samples	No field collected samples were used in the study
Ethics oversight	All animal care and used protocol were approved by IUCAC at Harvard Medical School

Note that full information on the approval of the study protocol must also be provided in the manuscript.
